# The impact of intellectual disability nurse specialists in the United Kingdom and Eire Ireland: An integrative review

**DOI:** 10.1002/nop2.690

**Published:** 2020-12-09

**Authors:** Jennifer Bur, Karen Missen, Simon Cooper

**Affiliations:** ^1^ School of Nursing and Health Professionals Federation University Churchill Vic Australia; ^2^ School of Nursing and Health Professions Federation University Churchill Vic Australia

**Keywords:** intellectual disability, Ireland, nurse specialists, nurses, nursing, review

## Abstract

**Aim:**

To identify and evaluate the impact of Intellectual Disability Nurse Specialists person‐centred care for people with intellectual disability.

**Design:**

An Integrative review of the literature was performed between January 2007–December 2017.

**Methods:**

Searching the PubMed Library of Medicine, Cumulative Index of Nursing and Allied Health Literature (CINAHL), Medline Ovid, PsychINFO, Health Source: Nursing/Academic edition. A total of eight articles were selected for the final study example, including four mixed methods studies and four qualitative studies.

**Results:**

Three Intellectual Disability Nurse Specialist models were evaluated, and three main themes emerged: person‐centred care, organizational and practice development.

**Conclusion:**

The Intellectual Disability Nurse Specialist expert knowledge and skills contribute to the development of effective systems and processes. The results highlighted the complex nature of the Intellectual Disability Nurse Specialist role and the importance of ongoing development, promotion and evaluation and their contribution to care in the healthcare setting.

**What does this paper contribute to the wider global clinical community?**

- This paper highlights the dearth of research into the Intellectual Disability Nurse Specialists’ complex role caring for people with Intellectual Disability in the general healthcare settings.
- People with intellectual disability have a lifespan significantly less than the general population and experience vulnerabilities when accessing health care. The Intellectual Disability Nurse Specialists person‐centred model of care ensures people with Intellectual Disability are central to their healthcare journey.
- More needs to be known about the experiences of people with Intellectual Disability accessing health care and the impact of Intellectual Disability Nurse Specialists’ in acute and general care settings to develop strategies for delivering high‐quality, sustainable care to this vulnerable patient population.

## 1 INTRODUCTION

People with intellectual disability have been identified as the most vulnerable and disadvantaged cohort in society, reliant on an under‐skilled medical workforce and with poor access to health services (Iacono et al., 2014; Troller et al., 2017). This cohort have a higher incidence of hospital stays and experience poorer health status attributed to Health Professionals stigmatizing attitudes, lack of understanding, misdiagnosis and poor communication (Department of Families & Community Services NSW, 2012; Iacono et al., 2014; Mencap, 2004, 2007). It is recognized that this cohort access health care at rates 8.7 times greater than the general population and represents 1% of the overall world‐wide population (Balogh et al., 2016; Department of Families & Community Services NSW, 2012; Troller et al., 2017). Moreover, this cohort have a life expectancy 20 years less than the general population which underscores the significant health inequalities experienced by this vulnerable group (Heslop et al., 2014; Troller et al., 2017).

International health priorities are influenced by the 2006 United Nations Convention on the Rights of Persons with Disability, that cemented the most extensive recognition of human rights (United Nations, 2018). Article 25 states that people with disability have the “right to enjoy the highest attainable standard of health without discrimination on the basis of disability” (United Nations & p^18^, ^2018^). This coupled with the World Health Organization Global Disability Action Plan 2014–2021 mandate that “international and national partners build understanding and promote importance of inclusion of disability (including rights in the curricula of schools of medicine, nursing and other healthcare professions)” (World Health Organisation & p^14^, ^2014^). The World Health Organization charges all countries to adhere to the ethical principle of person‐centred care that underpins the delivery of health care (World Health Organisation, 2015). Internationally and Nationally, nursing health priorities mandate optimal health care for people with intellectual disability, yet evidence suggests that targets are not being met (Australian College of Nursing, 2016; Dempsey, 2009; McCarron et al., 2018; Nursing & Midwifery Board of Ireland., 2020; Royal College of Nursing, 2016).

The United Kingdom Mencap, 2004 “Treat me right?” and Mencap, 2007 “Death by Indifference” reports identified people with intellectual disability who experienced healthcare inequalities leading to premature preventable death (Mencap, 2004, 2007). These reports launched changes in the United Kingdom healthcare sector for people with intellectual disability, with the introduction of the acute healthcare Learning Disability Liaison Nurse. Alternatively, the Éire Ireland Health Service Executive commissioned a national project to shape the future role of Intellectual Disability Nursing in Ireland (McCarron et al., 2018). This report has set out a clear direction for future intellectual disability nursing ensuring the best possible health and social care delivery to people with intellectual disability throughout their lifespan (McCarron et al., 2018).

Currently, Intellectual Disability Nurse Specialists represent 2.4% of the UK nursing workforce and 5.7% of the Éire Ireland nursing workforce (McCarron et al., 2018; Royal College of Nursing, 2020). The UK consists of three Great Britain countries [England, Scotland and Wales] and Northern Ireland, have two Intellectual Disability Nurse Specialist models; the community‐based Learning Disability Nurses and the acute healthcare sector Learning Disability Liaison Nurses (RCN, 2020). In Éire Ireland, the Registered Nurse Intellectual Disability, Clinical Nurse Specialists and Advanced Nurse Practitioner provide specialist health care for people with intellectual disability (McCarron et al., 2018). The Intellectual Disability UK and Éire Ireland specialist nurse pre‐registration and postgraduate programmes are the only health profession specifically educated in intellectual disability; therefore, it is pertinent to evaluate their impact on person‐centred care (Doody, 2017). Compared with the Australian Nursing Baccalaureate, Intellectual Disability content is minimal which has an impact on the quality of acute healthcare delivery and health professional attitudes when caring for people with intellectual disability (Iacono et al., 2014; Troller et al., 2016). This integrative review will identify and evaluate the impact of Intellectual Disability Nursing Specialists person‐centred care for people with intellectual disability.

## 2 AIM AND METHODS

The objective of this review was to systematically identify, appraise and synthesize the best available evidence for the impact of Intellectual Disability Nurse Specialists in comparison with an alternative or no intervention. The PRISMA checklist was used as a guide to the search and reporting of the results (Moher et al., 2015) (Figure S1). This research method extracted data in a systematic and methodological rigorous standard, aiming to synthesize investigations to support professional decision‐making, improve clinical practice and reveal gaps in knowledge (Souza et al., 2010). A six‐phase search strategy was used for this integrative review (Souza et al., 2010). The review included primary qualitative and mixed methods study designs published in English, from January 2007–December 2017.

All citations and abstracts identified by the search strategy were downloaded to an Excel spreadsheet, and duplicates were identified and removed. Potential articles were screened for eligibility by title and abstract. Articles that met the inclusion criteria were independently reviewed by two authors. The articles included four qualitative studies: (Brown et al., 2016; Doody et al., 2013, 2016, 2017) and four mixed methods studies (Brown et al., 2012; Castles, Bailey, Gates, & Sooben, 2012, 2014; MacArthur et al., 2015).

### 2.1 Search strategy

A search conducted of the PubMed database identifying Medical Subject Headings. The following databases were accessed: Cumulative Index of Nursing and Allied Health Literature (CINAHL), Medline Ovid, PsychINFO, Health Source: Nursing/Academic edition. A systematic strategy based on the PICO mnemonic used derivatives of “People with Intellectual OR Developmental OR Learning AND Disability” (Population); “Impact of Intellectual OR Developmental OR Learning OR Clinical Nurse Specialist AND Disability Nurse” (Intervention); “General Population” (Comparison); and “Evaluation OR Effectiveness OR Person‐centred care in the healthcare sector” (Outcome). Lastly, the reference lists of recent studies and reviews were searched for eligible papers that may have been previously missed.

### 2.2 Eligibility criteria

The inclusion criteria established for articles selected were full texts available in the selected databases between 2007 and 2017 published in English; primary studies with qualitative, quantitative and mixed method study designs. The year 2007 was chosen as it coincided with the emergence of literature describing the size, extent and nature of learning disability nursing research (Griffiths et al., 2009). Peer‐review primary research articles investigating the effectiveness of intellectual disability nursing models and determining their impact on person‐centred care delivery to individuals with Intellectual Disability and their families.

### 2.3 Critical appraisal

The methodological quality of each study was assessed using two critical appraisal tools developed by Critical Appraisal Skills Programme [CASP]: Qualitative Assessment and Review Instrument for qualitative studies (Critical Appraisal Skills Program, 2014) and Health Care Practice Research and Development Unit (HCPRDU): Mixed Methods Study Design (Long et al., 2002). An extraction form was used to extricate relevant data comprises of ten domains: aims, methodology, study design, recruitment, data collection, researcher critical analysis, ethical consideration, rigorous data analysis, findings clearly stated and research value, with a potential score range to 100 per cent (Bettany‐Saltikov & McSherry, 2016). The scores are listed in Table S1. A further independent content analysis undertaken by the authors to analyse the outcomes of each article for data that specifically answered the aim of this review.

## 3 RESULTS

### 3.1 Study selection

The search produced a total of 189 articles. The major headings and abstracts were scrutinized for relevance with 172 abstracts excluded in accordance with the inclusion criteria, and duplications were removed. The abstracts of the remaining 15 articles were scrutinized for relevance, by three reviewers, and read to identify eligibility applying the inclusion criteria. If there were conflicting opinions about inclusion/exclusion, the paper was discussed with supervisors (SC & KM) and the inclusion and exclusion criteria were re‐applied.

This left ten research articles for further detailed assessment by two supervisors (SC & KM). The author forwarded a spreadsheet detailing research articles; title, abstract, year, reference; and study type to supervisors (SC & KM) for further scrutiny. Further two articles were removed, leaving eight articles that met all inclusion criteria. After the study selection process, eight studies were included in the integrative review.

### 3.2 Characteristics of included studies

The included studies were published between 2007 and 2017 and were conducted in two countries: six in the United Kingdom and two in Éire Ireland (see Table S1). Five of the eight studies evaluated the role of the Learning Disability Liaison Nurse (Brown et al., 2012, 2016; Castles et al., 2012, 2014; MacArthur et al., 2015); whilst one study evaluated the Registered Nurse—Intellectual Disability role (Doody et al., 2013); and two studies evaluated the Clinical Nurse Specialist—Intellectual Disability roles (Doody, Slevin, & Taggart, 2016, 2017).

### 3.3 Participants characteristics

Study sample sizes varied from 7 to 85 (mean = 38) participants (including Intellectual Disability nurses, carers, family and health professionals). Participants varied with two reporting on interviews with 23 family/carers and persons with intellectual disability (Brown et al., 2016; Doody et al., 2017); two studies reported on interviews with 38 individuals including Registered Nurse Intellectual Disability and Clinical Nurse Specialists in Intellectual Disability roles (Doody et al., 2013, 2016). The remaining four of the eight studies interviewed 242 family/carers, persons with intellectual disability and Healthcare professionals (Brown et al., 2012; Castles et al., 2012, 2014; MacArthur et al., 2015).

### 3.4 Theme Outcome analysis

From the eight peer review articles, three main themes emerged: (1) person‐centred care; (2) Systems and care co‐ordination; and (3) Practice Development—Professional and Client/Family education.

#### 3.4.1 Person‐centred care

All studies reported that person‐centred care was a significant component of the Intellectual Disability Nurse Specialists’ role (Brown et al., 2012, 2016; Castles et al., 2012, 2014; Doody et al., 2013, 2016, 2017; MacArthur et al., 2015). This was associated with the Intellectual Disability Nurse Specialist “seeing the person rather than the disability” and their “unique attitude regarding dignity, respect and personhood of the individual which is seen as the cornerstone of care” (p116), which played a significant role in achieving optimal outcomes for people with intellectual disability (Doody et al., 2013). Castles et al. (2014) reinforced this notion by stating that Intellectual Disability Nurse Specialists “are unique in being the only professional group prepared to work specifically with people with intellectual disability and are the profession to shape and address barriers in healthcare for people with intellectual disability” (p279), thus shaping and empowering people with intellectual disability to make informed decisions about their own healthcare delivery (MacArthur et al., 2015). Doody et al. (2017) recognized that Intellectual Disability Nurse Specialists empowered people with intellectual disability and their families through an inclusive person‐centred approach to all care, enabling attainment of rights and entitlements, advocacy, care planning and identifying resources (Doody et al., 2017).

The ability of Intellectual Disability Nurse Specialists to effectively communicate in relation to patient capacity is paramount in person‐centred care (Brown et al., 2016). Brown (2016) further acknowledged that the flexibility of the Learning Disability Liaison Nurse role, facilitated effective communication and information sharing, through actively advocating and mediating to improve communication between the person with intellectual disability, their family and carers and health professionals (Brown et al., 2016; Castles et al., 2012). Castles (2012) acknowledged that families were relieved when the Intellectual Disability Nurse Specialist provided patients with intellectual disability with augmented and alternative communication tools and trained ward staff on their use facilitating improved person‐centred care. The Intellectual Disability Nurse Specialists’ wealth of expertise and clinical experience of working with patients, with complex co‐morbidities and communication issues played a central role in facilitating communication, especially in relation to treatment consent and patient‐centred approaches (Brown et al., 2016).

#### 3.4.2 Systems and co‐ordination of care

All eight studies identified that Intellectual Disability Nurse Specialists promoted and facilitated effective systems and co‐ordination of care in the general and acute healthcare sectors through *reasonable and achievable adjustments* and policy and procedure development (Brown et al., 2012, 2016; Castles et al., 2012, 2014; Doody et al., 2016, 2017; MacArthur et al., 2015). The development of a referral service ensured that people with intellectual disability were linked with and able to access Intellectual Disability Nurse Specialist services (Brown et al., 2012; MacArthur et al., 2015). An emphasis on *reasonable and achievable adjustments* ensured advanced admission preparation including; securing additional nursing resources; preparing ward staff; facilitating equipment and communication tools; advanced discharge planning; transfer from acute hospital to continuing care environment for end‐of‐life care; single room or group room accommodation; and private waiting areas in outpatient departments (Brown et al., 2016; Castles et al., 2012, 2014; MacArthur et al., 2015). Intellectual Disability Nurse Specialists were found to advocate for people with intellectual disability by supporting medical teams to achieve equitable care and through treatment options based on clinical need, rather than intellectual disability (MacArthur et al., 2015). Intellectual Disability Nurse Specialists liaised with community care managers, hospital social services departments and discharge planners to assist with timely discharge (Castles et al., 2014) and worked directly with governmental services, provided greater autonomy and enabled fluid inter‐agency working between and across health and social services (Doody et al., 2016).

Intellectual Disability Nurse Specialists also ensured continuity of person‐centred care approaches through the development of policies, procedures and care pathways, specifically tailored to meet people with intellectual disability (Brown et al., 2012). This ensured access to National Screening programmes and quality indicator improvements tailored to intellectual disabled including palliative care checklists, maternity care pathways and transition care pathways from paediatric to adult services (Brown et al., 2012) Intellectual Disability Nurse Specialists were responsible for the development of the Disability Distress Pain Assessment tool, which provided hospital staff with an effective communication tool to identify distress cues in people with intellectual disability when they have severely limited communication (MacArthur et al., 2015; Northgate & Prudhoe NHS Trust Palliative Care Team & St. Oswald's Hospice, 2006).

#### 3.4.3 Practice development

Six of the eight studies identified that Intellectual Disability Nurse Specialists promoted and facilitated effective professional development and practice development for clients/family (Brown et al., 2012; Castles et al., 2012, 2014; Doody et al., 2012, 2016). Intellectual Disability Nurse Specialists supported and enabled a range of educational and professional development programmes for health professionals that contributed to the enhancement of knowledge, skills and attitudes in caring for people with intellectual disability (Brown et al., 2012). Education and training were facilitated through a range of programmes, such as hospital induction programmes, patient experience training, nurse preceptorship, junior doctors’ training, medical assessment unit training and healthcare support worker workshops (Castles et al., 2014; Doody et al., 2013, 2016). Also, a range of tailored training courses were developed and delivered that focused on effective communication, capacity and consent and the appropriate application of legislation to health professionals (Castles et al., 2014; Doody et al., 2013, 2016). Intellectual Disability Nurse Specialists also contributed to undergraduate education in local universities, whilst some offered practical placement, providing a wider learning experience for student nurses to apply theory to practice (Brown et al., 2012). Furthermore, Intellectual Disability Nurse Specialists provided a wide variety of education and training for nurses, students and others involved in caring for persons with intellectual disability through presentations, conferences, practice publications and research (Doody et al., 2013, 2016). The Intellectual Disability Nurse Specialists central role also included supporting staff with education, training and advice from phone‐based consultations (Doody et al., 2016).

Alternatively, Intellectual Disability Nurse Specialists promoted and facilitated effective client/family education by sourcing training and tailoring information that met the client and family's educational need (Doody et al., 2016, 2017). Their educational role included not only information delivery but also linking clients and families into courses and training that enhanced client autonomy (Doody et al., 2017). By tailoring education to meet the client's specific needs and interventions, the Intellectual Disability Nurse Specialist demonstrated and provided training and resources that provided valuable education and information to families (Doody et al., 2016, 2017). Intellectual Disability Nurse Specialists acknowledged that education/information provision was a major component in providing realistic expectations and planning to support clients and families’ long‐term goals (Doody et al., 2017).

## 4 DISCUSSION

The World Health Organization Global Disability Action Plan 2014–2021 objective 1.4 mandate the removal of barriers and improve access to health services and programmes (World Health Organisation, 2014). The UK and Éire Ireland Intellectual Disability Nurse Specialists are unique in being the only healthcare specialist to address healthcare barriers in people with intellectual disability and enabling access to health services (Castles et al., 2014; Doody, 2017). Research identified that Intellectual Disability Nurse Specialists are achieving person‐centred care through holistic care co‐ordination, as a result of knowing the person, empowering and inclusive care co‐ordination (Brown et al., 2016; Castles et al., 2014). The Intellectual Disability Nurse Specialists extensive knowledge and skill ensures tailored care that meet individual needs, whilst their “unique attitude regarding dignity, respect and personhood is the cornerstone of care” (p1117) are all aspects of person‐centred care (Doody et al., 2013; McCarron et al., 2018). Their mediation and advocacy improved communication between health professionals and people with intellectual disability (Brown et al., 2016; Castles et al., 2014). This was achieved by individually adapting communication approaches by balancing information suited to the client's capacity at any given stage in their care (Brown et al., 2016; Castles et al., 2014).

The Equality and Human Rights Commission “reasonable and achievable adjustments” include three categories: Adjustments to physical features, auxiliary aids and services and adjustments to policies and procedures (Equality & Human Rights Commission, 2016). MacArthur et al., (2015) identified a fourth category relevant to people with intellectual disability specific needs, being “behavioural and emotional adjustments.” Intellectual Disability Nurse Specialists are in a prime position to promote and facilitate effective systems and care co‐ordination through *reasonable and achievable adjustments*. The Intellectual Disability Nurse Specialists’ expertise and clinical experience of working with patients with intellectual disability, with complex co‐morbidities and communication issues, *play a central role in facilitating communication, especially in relation to consent for treatment and ensuring patient centred approaches’* (Brown et al., 2016). A Learning Disability Liaison Nurse service ensures three key elements of *reasonable and achievable adjustments*: “auxiliary aids and services”; “policy and procedure adjustments” and “behavioural and emotional adjustments” (MacArthur et al., 2015). Pointu et al. (2009) research into hospital length of stay for people with intellectual disability identified that prior to the introduction of the Learning Disability Liaison Nurse, people with intellectual disability had prolonged hospital stays, which had an impact on overall health outcomes, including mortality rates. Since the introduction of the Learning Disability Liaison Nurse there have been significant reductions in length of stays (Castles et al., 2014), thus reducing complications associated with long hospital stays morbidity and mortality rates (Pointu et al., 2009).

Further the World Health Organization Global Disability Action Plan 2014–2021 objective 1.5 mandate international and national partners include disability training in medicine and nursing curricula (WHO & p^14^, ^2014^). The UK and Éire Ireland Intellectual Disability Nurse Specialists are meeting the WHO Global Disability Action Plan requirements and are the only countries providing a specialist nursing pre‐registration and postgraduate programmes. As Australian authors we note that a comparison between Australia and International baccalaureate nursing curriculum identified significant variation in intellectual disability knowledge. Pelleboer‐Gunnink et al. (2017) report that inadequately educated health professionals who lack confidence and skills in treating people with intellectual disability remain a significant issue in Australia. Research into Australian baccalaureate nursing curriculum identified that over half (52%) of the Nursing schools offered no intellectual disability content (Troller et al., 2016). Of the Nursing schools that provided units of study that contained some auditable intellectual disability content, the average of 3.6 hr per unit of study was taught (Troller et al., 2016). In addition to this, research into acute care nurses’ experience of nursing patients with intellectual disability identified that they felt underprepared when caring for people with intellectual disability with communication concerns (Lewis et al., 2017). This research identified failure of hospitals and staff to meet the needs of those with intellectual disability, limited knowledge and skills of staff and hospital system failures to achieve “reasonable and achievable adjustments” to care (Iacono et al., 2014). These factors identify that Australian health professionals require further education and skill development relating to intellectual disability, both in the current healthcare sector and at university level, as outlined in the WHO Global Disability Action Plan 2014–2021. As Australian authors, we note that in Australia the baccalaureate of nursing would benefit from further development of intellectual disability curriculum and/or the development of postgraduate qualifications to enhance nurse's knowledge. This coupled with practice development hours in intellectual disability care organizations would be advantageous.

The UK and Éire Ireland Intellectual Disability Nurse Specialist implementation of “reasonable and achievable adjustments” has been achieving person‐centred care targets. The Australian acute healthcare sector would benefit from the development and implementation of an Intellectual Disability Nurse role to facilitate improvements to healthcare policy and procedure development and; mandatory professional development for all health professionals to improve current healthcare delivery based on the Australian College of Nursing's mandate of person‐centred care.

## 5 CONCLUSION

This paper reviewed eight studies reporting on the impact of Intellectual Disability Nurse Specialists to determine effectiveness of person‐centred care. Three Intellectual Disability Nurse Specialist models in the United Kingdom and Éire Ireland were identified. Central tenets of the role focus on person‐centred care, organizational and practice development. Research identified that United Kingdom and Éire Ireland Intellectual Disability Nurse Specialists are the only countries providing specialist nurse pre‐registration and postgraduate programmes achieving person‐centred care targets in their communities.

The Intellectual Disability Nurse Specialist expert knowledge and skills contribute to the development of effective systems and processes, thereby improving patient outcomes. The results from this integrative review highlight the complex nature of the Intellectual Disability Nurse Specialist role and the importance of ongoing development, promotion and evaluation and their contribution to care in the general healthcare setting.

## 6 LIMITATIONS

In searching for studies to include in the integrative review, we were only able to find studies based in the United Kingdom and Éire Ireland as they are the only countries providing specialist nurse pre‐registration and postgraduate programmes; therefore, the scope of nursing professional practice in several countries are not represented. Further, the qualitative research articles were limited to small focus groups and semi‐structured interviews, or a mixture of both that used self‐reporting measures to assess the effectiveness of the Intellectual Disability Nurse Specialist service being evaluated.

## CONFLICT OF INTEREST

None.

## Supporting information

Figure S1

Table S1

## Data Availability

The data that support the findings of this study are available from the corresponding author, (JB), upon reasonable request.
