# Supplementary material for: The impact of intellectual disability nurse specialists in the United Kingdom and Eire Ireland: An integrative review
Source: Nurs Open. 2020 Dec 9;8(5):2018–24. doi: 10.1002/nop2.690 (PMC8363355; doi:10.1002/nop2.690)
Supplement: Supplementary file 1 — Figure S1 [file NOP2-8-2018-s002.docx]

**Figure 1:** *Flow Diagram of study selection.*

Additional records identified through grey literature searching

(*n* = 2)

CINAHL, Medline, PsychINFO, Healthsource: Nurse / Academic Education.

(n = 187)

Identification

Articles excluded that did not meet inclusion criteria
(n = 172)

Records remaining after duplications and editorials removed (n = 187)

Screening

Articles excluded that did not meet inclusion criteria

(n = 7)

Full-text articles screened and assessed for eligibility
(n = 15)

Eligibility

Qualitative articles
(n = 4)

Mixed methods articles
(n = 4)

Total Selected Studies
(n = 8 )

Included
