# Supplementary material for: The impact of intellectual disability nurse specialists in the United Kingdom and Eire Ireland: An integrative review
Source: Nurs Open. 2020 Dec 9;8(5):2018–24. doi: 10.1002/nop2.690 (PMC8363355; doi:10.1002/nop2.690)
Supplement: Supplementary file 2 — Table S1 [file NOP2-8-2018-s001.docx]

| **Author/s, Year & Setting** | **Study design** | **Sample** | **Aim** | **Data Collection Tools** | **Main Findings** | **Overall Score** |
| --- | --- | --- | --- | --- | --- | --- |
| Brown, M., Chouliara, Z., MacArthur, J., McKechanie, A., Mack, S., Hayes, M. & Fletcher, J. (2016). | Qualitative | ID Patients (*n* = 5)  Family/paid Carers (*n* = 13) | To investigate the experiences of patients with ID, family and paid carers regarding the role of liaison nurses and the delivery of compassionate, person-centred care . | Focus groups (n=11) and semi-structured interviews (n=7) | ***Qualitative - Themes:***  *Communication and the interpersonal experiences:*   1. Vulnerability, presence and ‘the human interface’ 2. Information balance 3. Critical points and broken trust   *Systems and co-ordination of care:*   1. Roles and responsibilities 2. Managing multiple transitions 3. ‘Flagging up’ and communication   IDLN have a significant impact with the general hospital in enabling compassionate and person-centred care, thereby meet the needs of patients with ID and by providing support for their carers. | 70% |
| Brown, M., MacArthur, J., McKechanie, A., Mack, S., Hayes, M., & Fletcher, J. (2012). | Mixed Methods | ***Quantitative:***  Referrals (n=323)  ***Qualitative:*** (*n*=85)   - ID patients (*n*=5) - Carers (*n*=16) - Primary healthcare professionals (n=39) - Secondary healthcare professionals (n=19) - Liaison nurses (n=6) | To examine the impact and outcomes of four Learning Disability Liaison Nurse Services in south-east Scotland on the healthcare experiences of people with ID attending general hospital care. | ***Quantitative:***  Referrals over 18 months (July 2007 – December 2008).  ***Qualitative:***  Semi-structured Interviews and Focus groups | ***Qualitative - Themes:***  LDLN role impacted on three (3) key areas:   1. Clinical patient care   Learning Disability Liaison Nurse services were valued by stakeholders by achieving person-centred outcomes.   1. Education and practice development.   LN expert knowledge and skills had an important role in developing effective systems and process within general hospital settings.   1. Strategic organisational developments   Learning Disability Liaison Nurse services outcomes highlighted the importance of supporting and promoting LN services and the challenges in delivering the multifaceted elements of the role. | 66% |
| **Author/s, Year & Setting** | **Study design** | **Sample** | **Aim** | **Data Collection Tools** | **Main Findings** | **Overall Score** |
| Castles, A., Bailey, C., Gates, B., & Sooben, R. (2014). | Mixed Methods | ***Quantitative:***  Referrals (n=115)  ***Qualitative***: (*n*=36)   - ID patients (n=7); - Carers (*n*=17); - Ward staff (*n*=12). | To conduce service evaluation whether the implementation of a Learning Disability Liaison Nurse service improved patient and carer experience within an acute hospital setting | ***Quantitative:***  Referrals over 6 months (September 2011- February 2012)  ***Qualitative***:  Semi-structured Interviews | ***Quantitative:***  Referrals did not fall below initial referrals recorded.  ***Qualitative*** - ***Themes:***  *ID Patient:*   1. Understanding role 2. Enhance communication   *Carers:*   1. Enhanced communication 2. Holistic approach to care   *Ward staff*:   1. Understanding role 2. Staff Intellectual Disability lack of knowledge   Reasonable adjustments, mental capacity issues, dealing with carers needs and dealing with funding arrangements. LN activities highly valued, not only for patients but also valuable contribution to the acute hospital staff work. | 66% |
| Castles, A., Bailey, C., Gates, B., & Sooben, R. (2012). | Mixed Methods | ***Quantitative:***  Referrals (n=115)  ***Qualitative:*** (n=36)   - ID patients (n=7) - Family Carer (n=5) - Paid Carer (n=5) - Care Manager (n=1) - Senior Nurses (n=4) - Physiotherapist (n=1) - Discharge Planner (n=1) - Staff nurses (n=5) - Community LD Nurse (n=5) - Community LD support worker (n=1) | The evaluation aim is to establish whether:   1. The liaison nurse-led service benefits clients and if so, how. 2. The liaison service meets Carers’ needs. 3. Clients understand the service. 4. Clients think the liaison service improves their experiences of hospital. 5. Hospital staff became more confident to meet clients’ needs. | ***Quantitative:***  Referrals over 6 months (September 2011- February 2012)  ***Qualitative:***  Semi-structured interviews (*n* = 36) | ***Qualitative - Themes:***  1. Liaison Nurse solved communication difficulties.  2. LN relieved communication stress with health professionals and translated family’s views into medical language.  3. Liaison Nurse bridged the gap between community services and the hospital. | 70% |
| **Author/s, Year & Setting** | **Study design** | **Sample** | **Aim** | **Data Collection Tools** | **Main Findings** | **Overall Score** |
| Doody, C. M., Markey, K., & Doody, O. (2013). | Qualitative | Registered Intellectual Disability Nurses (n=7) | To explore the experiences of Registered Intellectual Disability Nurses caring for the older person with intellectual disability. | Semi-structured (60-90 minute) interviews: | ***Qualitative - Themes:***   1. Care delivery 2. Teamwork 3. Proactive Planning 4. Family involvement in care 5. Inclusiveness 6. Inclusion 7. Attitudes to clients with ID 8. Services for older people with ID 9. Client focused care 10. Individualised care 11. Knowing the person 12. Best practice | 70% |
| Doody, O., Slevin, E., & Taggart, L. (2016). | Qualitative | Clinical Nurse Specialist – Intellectual Disabilities (n= 31) | To explore the contribution of clinical nurse specialists in intellectual disability nursing in Ireland. | Focus Groups [n=5]  (75-95 minutes) | ***Qualitative - Themes:***   1. Client care 2. Family care 3. Staff support 4. Service support 5. Community support 6. Supporting other agencies | 70% |
| Doody, O., Slevin, E., & Taggart, L. (2017). | Qualitative | Parents of people with Intellectual Disabilities (n=10) | The aim is to explore families’ perceptions of the contribution of clinical nurse specialists in Ireland. | Individual semi-structured interviews (38-65 minutes) | ***Qualitative - Themes:***   1. Personal Caring 2. Support 3. Liaison 4. Education and knowledge 5. Empowering | 70% |
| **Author/s, Year & Setting** | **Study design** | **Sample** | **Aim** | **Data Collection Tools** | **Main Findings** | **Overall Score** |
| MacArthur, J., Brown, M., McKechanie, A., Mack, S., Hayes, M., & Fletcher, J. (2015). | Mixed Methods | ***Quantitative****:*  Referrals (n=323)  ***Qualitative:*** (n=85)   - ID patients (*n*=5); - Carer (*n*=16); - Primary Care (*n*=39); - General Hospital (*n*=19); - Liaison Nurse (*n*=6); - Doctors (*n*= 7); - Hospital Consultant (*n*=4); - General Practitioner (*n*=3) | To examine the role of Learning Disability Liaison Nurses in facilitating reasonable and achievable adjustments to support access to general hospital services for people with learning disabilities. | ***Quantitative:***  Referrals over 18 months (September 2008- March 2010)  ***Qualitative:***  Semi-structured interviews and  Focus groups. | ***Qualitative - Themes:***  Reasonable adjustments:   1. Discharge planning; 2. Risk management; 3. Behavioural advice; 4. Communication advice; rearrangement of hospital appointment; 5. Eating and drinking guidelines; and 6. Arranging pre-admission visit to ward.   Family/formal Carers:  LN promoted understanding, safety, comfort and reduction in anxiety.  Carers:  LN influencing adjustment of outpatient appointment times and waiting areas.  Primary Care staff:  LN emphasis on reasonable adjustments ensuring appropriate hospital care and admission preparation.  General Hospital staff:  LN support reasonable adjustments in securing additional nursing resource, advanced admission preparation, using Disability Distress Assessment Tool [DisDAT] for assessment and communication. | 66% |
